# Recurrent Attacks of Zymotic Disease in the Same Person

**Published:** 1883-08

**Authors:** 


### Recurrent Attacks of Zymotic Disease in the Same Person.

Amongst the poor arguments to which fanatical people are driven to make a case against vaccination, the occurrence of small-pox in those who have been vaccinated is one. They generally omit to state that when this does happen the mortality and severity of the disease are immensely reduced. They also omit to state that people who have been well vaccinated twice have almost an absolute immunity from the dreaded disease. But though, for practical purposes, the rule is absolute, there must almost necessarily be an occasional exception, when we remember the well-known fact that some people have a liability to one or other of the zymotic diseases altogether peculiar and amounting to a veritable idiosyncrasy. The *Medical Record* of New York, in a recent number, contains the report of a case by

Dr. C. L. Dana, in the Practitioners' Society of New York, of a medical friend who before the age of twenty-one had suffered from five attacks of scarlet fever. In each attack the symptoms were well-marked; desquamation and, in most of the attacks, albuminuria were present. We have recently seen a case of a second attack of scarlet fever with well-marked desquamation. Not long ago an instance was brought under our notice of a man who had had two attacks of small-pox in twelve months, and—tell it not to Mr. P. Taylor—a history of one or two unsuccessful attempts at revaccination. If small-pox itself will not secure such people immunity, it is small blame to vaccination that it does not, though thorough vaccination in infancy and at puberty would probably be more likely to do so. Such people are to be excluded from all general arguments. They are as exceptional in their physical constitution as some of our anti vaccination friends are in their intellectual organization.—*The London Lancet.*

